# Anatomy and development of the larval nervous system in *Echinococcus multilocularis*

**DOI:** 10.1186/1742-9994-10-24

**Published:** 2013-05-04

**Authors:** Uriel Koziol, Georg Krohne, Klaus Brehm

**Affiliations:** 1 University of Würzburg, Institute of Hygiene and Microbiology, Josef-Schneider-Strasse 2, Würzburg, D-97080, Germany; 2 Department of Electron Microscopy, University of Würzburg, Würzburg, Biocenter, D-97078, Germany; 3 Universidad de la República, Facultad de Ciencias, Sección Bioquímica y Biología Molecular, Iguá 4225, Montevideo, CP 11400, Uruguay

**Keywords:** *Echinococcus*, Metacestode, Protoscolex, Nervous system, Neuropeptide, Serotonin, Acetylated tubulin

## Abstract

**Background:**

The metacestode larva of *Echinococcus multilocularis* (Cestoda: Taeniidae) develops in the liver of intermediate hosts (typically rodents, or accidentally in humans) as a labyrinth of interconnected cysts that infiltrate the host tissue, causing the disease alveolar echinococcosis. Within the cysts, protoscoleces (the infective stage for the definitive canid host) arise by asexual multiplication. These consist of a scolex similar to that of the adult, invaginated within a small posterior body. Despite the importance of alveolar echinococcosis for human health, relatively little is known about the basic biology, anatomy and development of *E. multilocularis* larvae, particularly with regard to their nervous system.

**Results:**

We describe the existence of a subtegumental nerve net in the metacestode cysts, which is immunoreactive for acetylated tubulin-α and contains small populations of nerve cells that are labeled by antibodies raised against several invertebrate neuropeptides. However, no evidence was found for the existence of cholinergic or serotoninergic elements in the cyst wall. Muscle fibers occur without any specific arrangement in the subtegumental layer, and accumulate during the invaginations of the cyst wall that form brood capsules, where protoscoleces develop. The nervous system of the protoscolex develops independently of that of the metacestode cyst, with an antero-posterior developmental gradient. The combination of antibodies against several nervous system markers resulted in a detailed description of the protoscolex nervous system, which is remarkably complex and already similar to that of the adult worm.

**Conclusions:**

We provide evidence for the first time of the existence of a nervous system in the metacestode cyst wall, which is remarkable given the lack of motility of this larval stage, and the lack of serotoninergic and cholinergic elements. We propose that it could function as a neuroendocrine system, derived from the nervous system present in the bladder tissue of other taeniids. The detailed description of the development and anatomy of the protoscolex neuromuscular system is a necessary first step toward the understanding of the developmental mechanisms operating in these peculiar larval stages.

## Introduction

The metacestodes of *Echinococcus multilocularis* and *Echinococcus granulosus* are the causative agents of alveolar and cystic echinococcosis (AE, CE), respectively. These larvae develop from onchospheres after oral ingestion of infectious eggs by their natural intermediate hosts (rodents for *E. multilocularis* and different ungulate species for each lineage of the *E. granulosus* species complex), but humans can also serve as accidental intermediate hosts. Most commonly, metacestode growth occurs in the liver (especially in the case of *E. multilocularis*), but other parenteral sites can also be infected. AE is one of the most dangerous zoonotic diseases in the Northern Hemisphere, while the more widely distributed CE is a neglected tropical disease of both medical and veterinary importance [1].

*Echinococcus* metacestode larvae consist of fluid-filled cysts, that are covered by an acellular laminated layer. Within the cysts, the cells are organized as a thin layer, the germinal layer or “germinal membrane”, that is juxtaposed to the laminated layer. This layer consists of several cell types such as tegumental cells, which compose the tegumental syncitium that covers the cyst’s interior and secretes the laminated layer. Other cell types have been described within the germinal layer, including glycogen storing cells, calcareous corpuscle cells, cells forming the excretory ducts (and in the case of *E. granulosus* also flame cells), muscle cells and undifferentiated stem cells [2-5]. In the case of *E. granulosus*, each larva grows typically as a single cyst, whereas the *E. multilocularis* metacestode proliferates as a labyrinth of small interconnected vesicles, that infiltrates the tissues of the host.

Within the cysts, brood capsules are formed from the germinal layer. These are vesicular structures, with a cavity lined by a tegumental syncytium (that is, with the opposite polarity to that of the cyst wall, in which the tegumental syncytium covers the external side). Two theories have been proposed for the mechanism of formation of the brood capsules, based on histological and electron-microscopical investigations: that they are formed by cavitation of a solid mass of cells that accumulates as a bud in the germinal layer [2,6], or by invagination and posterior constriction of a thickened region of the germinal layer [7,8].

Within the brood capsules, protoscoleces are formed, which are the infective stage for the definitive hosts (canids). The protoscolex contains a scolex with four suckers and an armed rostellum, already very similar in morphology to the scolex of the adult worm. The scolex is invaginated, but after ingestion by the definitive host the scolex evaginates and the protoscolex establishes in the intestine, were it develops into the adult worm. The formation of protoscoleces within the brood capsule has been described by several authors [6-10]: initially, a small bud develops on the inner side of the brood capsule wall, covered by the brood capsule tegument, and grows into the brood capsule cavity. Early during development, the rostellar primordium can be distinguished at the tip of the protoscolex bud, and later the primordia of the suckers become apparent. Once the development of the rostellum and the suckers is complete, the scolex invaginates into the posterior body, which is now only connected to the brood capsule by a thin stalk (however, the invagination of the scolex before development is completed has also been described [8,11]). The formation of multiple protoscoleces within each cyst results in asexual multiplication in the intermediate host, a process that is rare in cestodes but relatively common within the family Taeniidae (genera *Taenia* and *Echinococcus*[11-15]).

Few ultrastructural, histochemical or immunohistochemical studies have been conducted on the nervous system of *Echinococcus*, resulting in a rather incomplete picture of its morphology and development. The elements of the nervous system have been best described for the adult form of *E. granulosus*, by acetylcholinersterase histochemistry (AChE HC) [16] and by immunohistofluorescence (IHF) against 5-hydroxytryptamine (serotonin; 5-HT) [17], with similar but also complementary results (not all elements could be described with each technique). Briefly, in the scolex, it consists of two main lateral ganglia connected to each other by transverse and ring commisures, two postero-lateral ganglia, and a rostellar nerve ring and paired rostellar ganglia. Ten longitudinal nerve cords run in the strobila, that are interconnected by numerous transverse commisures. Neurites emanate from the nerve cords forming a subtegumental plexus innervating the subtegumental musculature. In the protoscolex of *E. granulosus*, the nervous system was partially described by a pioneering IHF study using antibodies against 5-HT and against mammalian neuropeptides [18]. It was suggested that the nervous system was similar but simpler than that of the adult worm, with fewer 5-HT positive cells, and lacking for example the postero-lateral ganglia. No detailed IHF study has been performed with other neural markers for protoscoleces of *Echinococcus* spp., no study has been conducted in *E. multilocularis* and to the best of our knowledge, there has been no study on the cyst wall of the metacestode.

In this work, we use phalloidin staining and IHF with antibodies against several nervous system markers to describe the muscle and nervous systems in the germinal layer and protoscolex of *E. multilocularis*. We provide evidence for the first time of nerve cells in the germinal layer of the cyst wall, and describe the development of the muscle and nervous systems of the protoscolex during asexual multiplication. Finally, we provide the most detailed account of the neuroanatomy of the mature protoscolex available to date.

## Results

### The muscle system in the cyst wall

**Figure 1:**
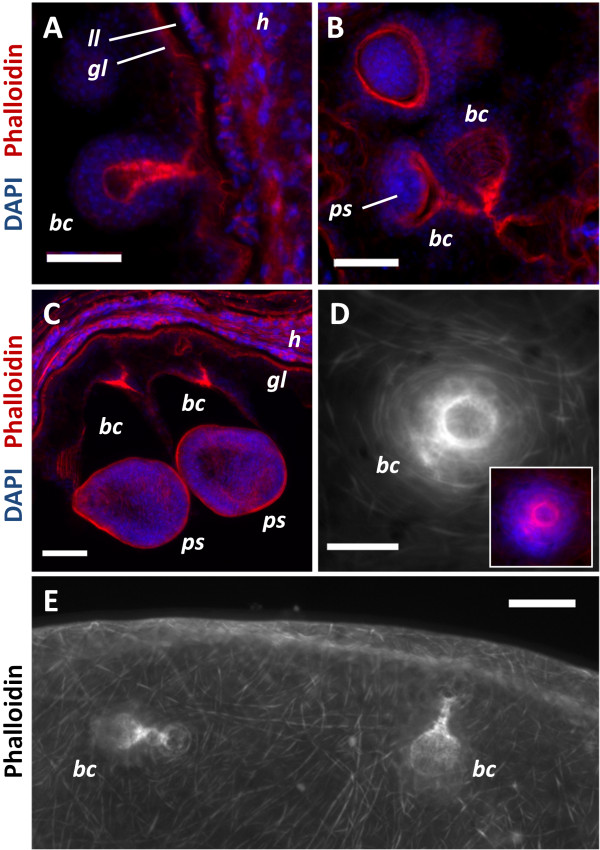
**Muscle fibers in the germinal layer and during brood capsule formation.** All specimens stained with Phalloidin. **A**. Early brood capsule development (*bc*, brood capsule; *gl*, germinal layer; *h*, host tissue; *ll*, laminated layer). **B**. Brood capsules containing protoscolex buds (*ps*). **C**. Brood capsules during late protoscolex development. **D**. Whole-mount preparation showing an early brood capsule seen from above (inset shows merge with DAPI staining). Note the organization of the muscle fibers in the brood capsule region. **E**. General arrangement of the muscle fibers in the metacestode and brood capsules. **A**, **B** and **C** are sections from *in vivo* cultured material; **D** and **E** are whole-mount preparations of *in vitro* cultured material. Bars represent 50 μm.

**Figure 2:**
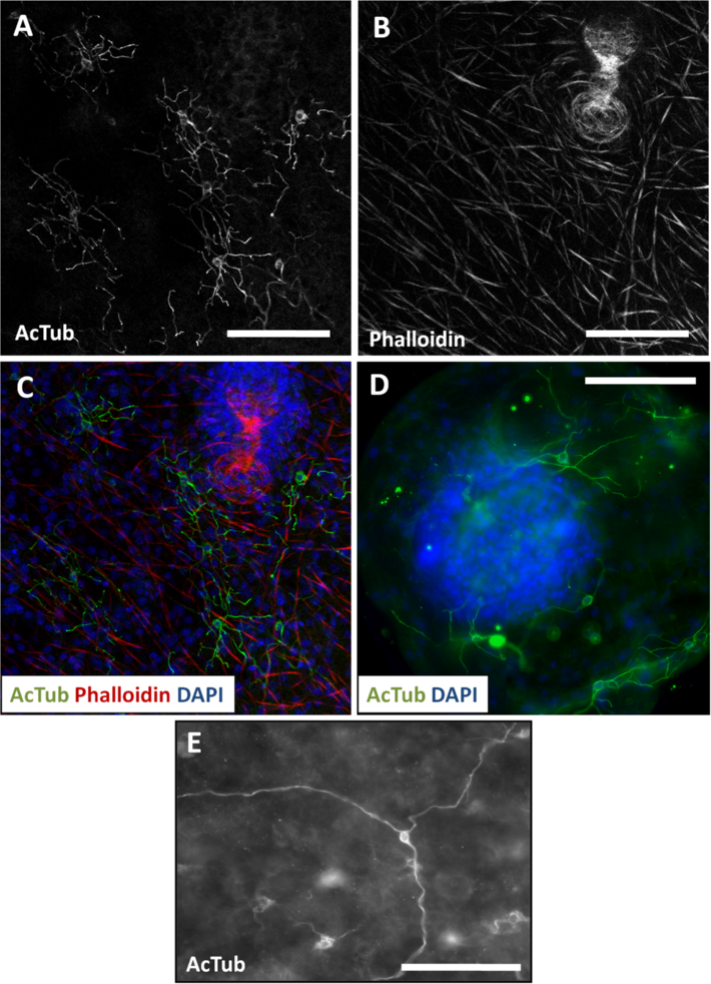
**AcTub-IR in whole-mount preparations of the germinal layer. A**. AcTub-IR nerve cells in the germinal layer (*in vitro* cultured cyst, analyzed by confocal microscopy). **B**. Corresponding image of Phalloidin staining. **C**. Merge of AcTub-IR and Phalloidin staining combined to DAPI staining. Notice the lack of AcTub-IR cell bodies in the brood capsule. **D**. AcTub-IR nerve cells in a very small vesicle obtained from *in vivo* culture, containing only one brood capsule with a protoscolex bud (this image is a mosaic of three pictures and an overlay of several focal plains of epifluorescence microscopy). **E**. Unusual AcTub-IR nerve cell with long, unbranched neurites (*in vitro* cultured material). Neurites from other nerve cells are outside of the focal plane. Bars represent 50 μm.

**Figure 3:**
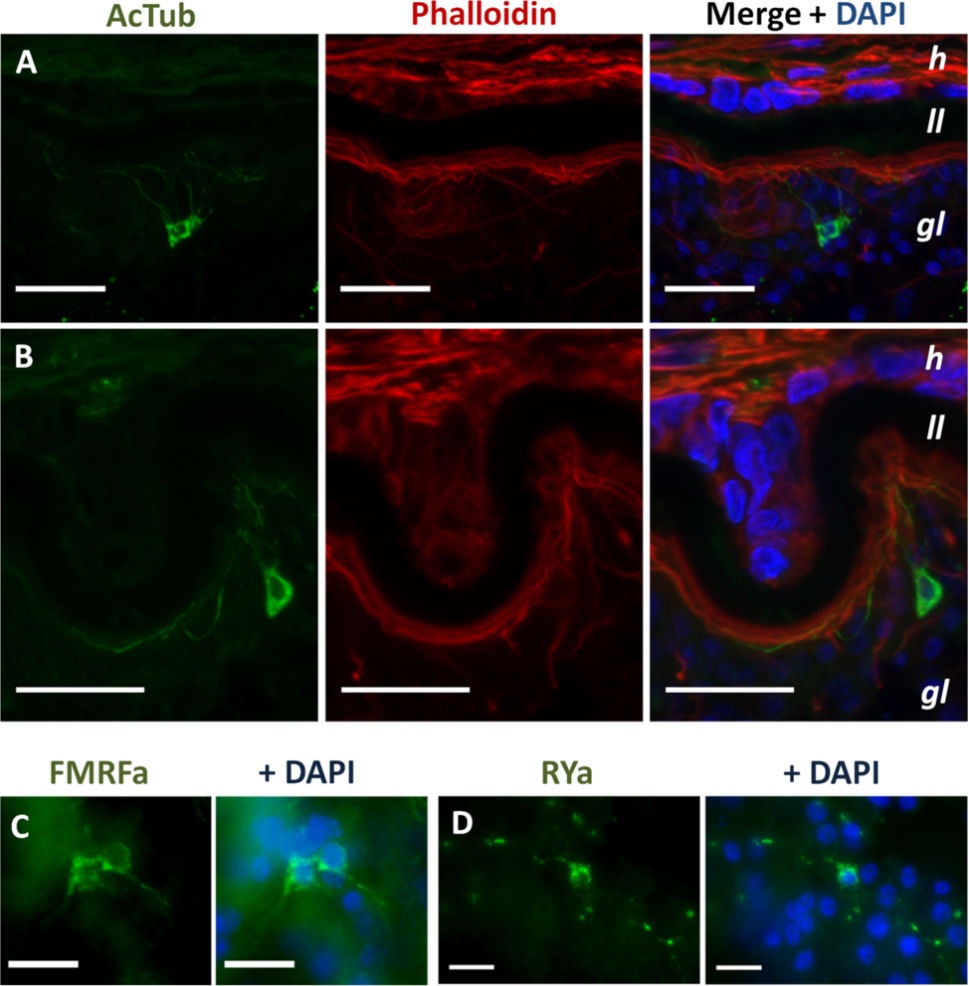
**AcTub-IR, FMRFa-IR and RYa-IR in sections of the germinal layer. A**. multipolar AcTub-IR cell. Note the neurites projecting towards the subtegumental muscle layer. **B**. Pseudounipolar AcTub-IR cell. **C**. FMRFa-IR in two cell bodies and their projections. **D**. RYa-IR in a nerve cell body and projections in a tangential section of the germinal layer. **A** and **B** are confocal microscopy images from *in vivo* cultured material; **C** and **D** are epifluorescence images from *in vitro* cultured material. Abbreviations are as in Figure 1. Bars represent 20 μm in A, B and 10 μm in **C**, **D**.

Phalloidin staining revealed long muscle fibers in the metacestode cyst wall, situated below the tegument, external to all the nuclei of the cells of the germinal layer (subtegumental muscle layer) (Figures 1, 2 and 3). The fibers show no particular arrangement at a global level, but locally bundles of parallel fibers can be seen. Muscle fibers had been previously described by Transmission Electron Microscopy (TEM) in the cyst wall of both *E. granulosus* and *E. multilocularis*[2-4]. Larger vesicles show a denser array of fibers and fiber bundles, especially in the case of *in vitro* cultured vesicles, whereas smaller vesicles show scarcer, isolated fibers. Fully mature vesicles obtained *in vivo* become packed with protoscoleces and calcareous corpuscles, with a very thin germinal layer that has few or no muscle fibers (data not shown).

### The nervous system in the cyst wall

Acetylated tubulin-α immunoreactivity (AcTub-IR) in the cyst wall revealed a discontinuous nerve net of multipolar cells, with perykarya sunken in the deeper levels of the germinal layer, and long branching neurites projecting towards the subtegumental muscle layer, in many cases contacting the neurites from other nerve cells (Figures 2 and 3A, 3B). Also, a smaller number of other AcTub-IR nerve cells were found, with longer and much less branched projections (Figure 2E). The neurites of these cells run deeper in the germinal layer, below most of the subtegumental muscle fibers.

The AcTub-IR nerve net was found in vesicles from both *in vivo* and *in vitro* cultures, being most developed in larger vesicles with a well developed muscle layer and a thicker germinal layer, while in smaller vesicles fewer nerve cells were apparent. Upon *in vitro* cultivation, the nerve net was clearly observable in all vesicles, distributed throughout the cyst wall. In *in vivo* generated material, the nervous system was observable in some but not all the vesicles. Particularly, degenerating and fully mature vesicles, with a thin germinal layer, showed few or no nervous elements. Also, in very young and small vesicles, with a thin germinal layer [6,10], muscle fibers were sometimes not apparent, and the nerve cells were few, distributed in small patches in the cyst wall. From whole-mount preparations of in vitro cultured material, we estimated that AcTub-IR nerve cells represented 0.2% to 1.9% of all cells in the germinal layer.

The only cells labeled in the cyst wall with the antibody against AcTub were the nerve cells and some very rare flame cells, which were only observed in material from *in vivo* cultivation. These are easily distinguishable from the nerve cells given their morphology (including an AcTub-IR bundle of cilia or "flame", and a roundish cell body that is also strongly AcTub-IR), and by their co-staining with phalloidin in a basal ring surrounding the bundle of cilia [19,20]. Their morphology is however somewhat abnormal as compared to the morphology of flame cells in the protoscolex, since the phalloidin ring and the bundle of cilia are small and irregularly shaped [Additional file 1]. AcTub-IR has also been described in gland cells and glandular ducts in other flatworms (see for example [21]), but because of the morphology of the AcTub-IR cells in the cyst wall, we conclude that most, if not all, the AcTub-IR positive cells that are not flame cells correspond to nerve cells.

A small subpopulation of the cells in this nerve net was revealed by FMRFa immunoreactivity (FMRFa-IR), that was localized to a few nerve cells extending long, varicose projections with little branching (Figure 3C). Staining was seen in the nerve cell bodies and in the varicose neurites. Similarly, RYamide immunoreactivity (RYa-IR) and FVamide immunoreactivity (FVa-IR) was revealed in even smaller numbers of nerve cells with similar morphology, and in many cases only the nerve projections were apparent, the perykarya being indistinct (Figure 3D and data not shown).

Interestingly, no clear evidence was obtained for the existence of serotoninergic or cholinergic nerve cells, although 5-HT and acetylcholine are two of the best characterized classical neurotransmitters in cestodes [22], and both kinds of elements are detectable in the protoscolex ([18]; see below). Only some (presumably non-specific) 5-HT-immunoreactivity (5-HT-IR) was observed surrounding the calcareous corpuscles (data not shown). Furthermore, no acetylcholinersterase activity was observed in the nerve cells by HC. Only some very rare cells, apparently lacking projections, were positive for acetylcholinesterase HC, and these were all negative for AcTub-IR [Additional file 2].

The finding of a nervous system in the cyst wall was unexpected, since it is a non-motile stage, and a nervous system has never been described in previous ultrastructural studies on *Echinococcus*[3-5]. Therefore, we performed our own TEM studies with *in vitro* cultured vesicles [Additional file 3A, B]. In sections tangential to the cyst wall, we found long, thin cellular projections, packed with microtubuli, and in some cases containing mitochondria and some few small electron-dense vesicles. We interpret these as the projections of nerve cells. They are few, small, and rather inconspicuous, possibly explaining why they have not been previously described. We have found in rare occasions cell bodies connected to such projections, which we identify as the putative nerve cells, containing cytoplasm with numerous free ribosomes and mitochondria, and with regions packed with microtubuli in parallel arrays and occasional vesicles [Additional file 3C].

### Formation of brood capsules

Brood capsule formation begins as a compact, bud shaped accumulation of cells in the germinal layer. By confocal microscopy of whole-mounts and sections, we were able to clearly confirm that the brood capsule forms from an invagination of the germinal layer that is not accompanied by the laminated layer [7] (Figure 1) [Additional files 4, 5 and 6]. Furthermore, there is a reorganization of the subtegumental muscle layer, as muscle fibers with a circular disposition accumulate surrounding the invagination. Later, the base narrows, forming a stalk that connects the brood capsule proper to the germinal layer, but even in this and later stages this stalk is hollow and internally covered by muscle fibers, that are predominantly circular. The other end becomes dilated, with the walls becoming thinner. Thus, the subtegumental layer of muscle fibers in the cyst wall is continuous with the muscle layer in the brood capsule stalk and wall. The muscle fibers in the dilated brood capsule do not initially show any particular arrangement, but when the protoscolex bud emerges as a thickening in the brood capsule wall, the muscles become rearranged, showing a clear grid of longitudinal and circular muscles that extend from the brood capsule into the developing protoscolex (see also below). The rest of the brood capsule becomes a thin layer of germinal tissue as protoscolex development proceeds, with sparser and less organized muscle fibers.

We found no evidence of nerve cell bodies within the brood capsule itself. However, AcTub-IR perykarya were seen at the base of some brood capsules, and projections from the nerve cells in the cyst wall sometimes entered the base of the early brood capsules. No connection was observed between the nervous system of the cyst wall and the developing protoscolex.

### The muscle system of the mature protoscolex

**Figure 4:**
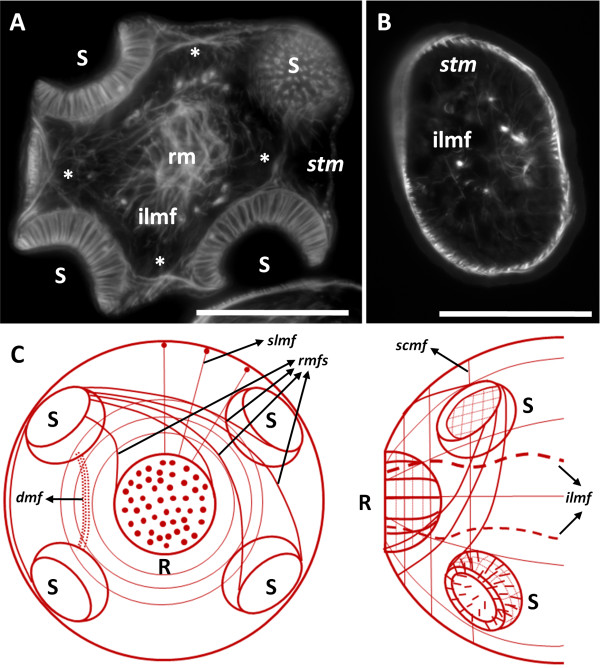
**The muscle system of the protoscolex. A**. Phalloidin staining of a transverse section of the scolex. **B**. Phalloidin staining of a transverse section of the posterior body. **C**. Diagram showing the main groups of muscle fibers in the scolex (left, frontal view; right, dorsal view). See the main text for details. Abbreviations: *dmf*, deep muscle fibers connecting dorso-ventrally adjacent suckers; *ilmf*, inner longitudinal muscle fibers; S, sucker; *slmf*, subtegumental longitudinal muscle fibers, *scmf*, subtegumental circular muscle fibers; *stm*, subtegumental muscles (referring to both longitudinal and circular muscle fibers); R, rostellum, *rm*, radial muscles below the rostellar pad; *rmfs*, radial muscle fibers connecting each sucker to the others and to the rostellum. Asterisks mark several groups of muscle fibers connecting the suckers to each other. Bars represent 50 μm.

**Figure 5:**
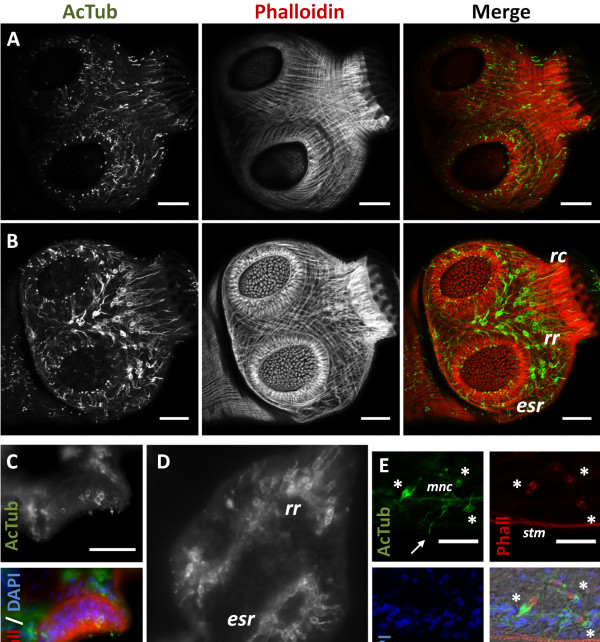
**AcTub-IR in the protoscolex. A**. AcTub-IR in the nerve terminals in the surface of the protoscolex (confocal microscopy, merge of 3 optical sections). **B**. AcTub-IR in the nerve cell bodies and neurites below the sections shown in **A** (confocal microscopy, merge of 3 optical sections). **C**. AcTub-IR cells and their projections in the internal sucker nerve ring (section). **D**. AcTub-IR cell bodies and their projections deep within the scolex (section). **E**. Detail showing a nerve terminal penetrating and traversing the tegument (arrow). Asterisks mark flame cells. (In panel BF, bright field microscopy is added to the merge of the fluorescence channels). Abbreviations: *esr*, external sucker ring; *mnc*, main nerve cords; *rc*, coronae of neurites projecting from the rostellar ring; *rr*, rostellar ring; *stm*, subtegumental muscle layer. Bars represent 25 μm in A, B, and D, 50 μm in **C** and 20 μm in E.

For the sake of clarity, we will first describe the muscular and neural anatomy of the mature protoscolex, as observed after evagination (Figures 4 and 5). The muscle system was described by classical histological methods for *E. granulosus* by Coutelen et al. [23], and our results are, with few exceptions, comparable to theirs, so the musculature will be discussed only briefly.

The subtegumental muscle layer consists of a grid of outer circular and inner longitudinal muscle fibers. The longitudinal muscle fibers traverse all along the anteroposterior axis, from the rostellum to the stalk and excretory pore, except for those fibers that are interrupted by the suckers. The circular fibers are found throughout the body except at the level of the suckers. Closely beneath the subtegumental muscles of the scolex, there are groups of muscle fibers projecting more or less radially from the margin of the suckers, connecting each sucker to the others and to the rostellum. Some of the muscles project from the antero-medial border of one sucker, crossing the midline towards the postero-medial border of the adjacent sucker on the same side of the scolex. Other muscle fibers emanating from the margin of the suckers, that are fixed more anteriorly, also cross the midline and connect each sucker to the diagonally opposite sucker, on the other side of the scolex, while still other fibers connect each sucker to the rostellum. Dorso-ventrally opposed suckers are connected by fibers emanating from the lateral margin of each sucker, but also by deep muscle fibers that are fixed to their bases.

Deep in the parenchyma, there is a group of few but prominent longitudinal fibers, connecting the rostellar pad to the posterior of the protoscolex. These muscles are probably associated with the invagination of the scolex.

The suckers themselves are complex muscular organs, with circular fibers both at the opening of the sucker, and also surrounding the wall of the sucker, forming a grid with longitudinally oriented fibers. On the outer face of the sucker, beneath the tegument, there is also a grid of longitudinal and traverse muscles. The most important group of muscle fibers in the sucker are the perpendicular fibers, connecting the base and the outer face of the sucker.

The rostellar pad, beneath the hooks, is similar in its constitution to the suckers, being surrounded at its outer wall by circular and longitudinal muscles, and with powerful perpendicular fibers connecting the base to the tip of the pad.

Finally, there are numerous muscle fibers in the scolex that are not so intricately organized, including dorso-ventral fibers and transverse fibers. In particular, there is a great accumulation of radial fibers beneath the rostellar pad proper. These could be of importance for the evagination of this organ. Few other fibers can be seen in the body of the protoscolex.

### The nervous system of the mature protoscolex

#### Patterns obtained with antibodies

**Figure 6:**
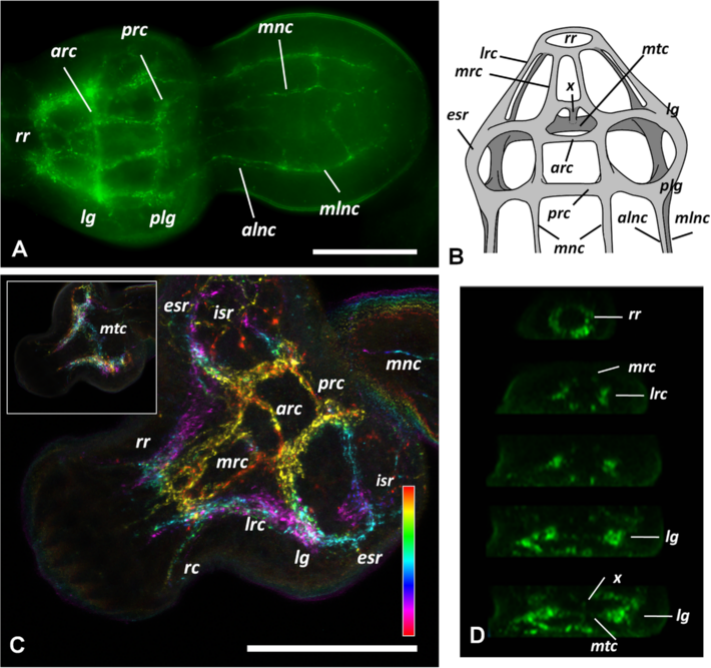
**FMRFa-IR in the protoscolex. A**. General view of the protoscolex; merge of several focal planes of epifluorescence microscopy, anterior is to the left. **B**. Diagram showing the main elements of the central nervous system in the scolex as identified by FMRFa-IR. **C**. Detail of FMRFa-IR in the scolex, confocal projection of 10 um of optical sections with color-coded depth; the inset shows another projection of 10 um thickness, deeper within the scolex, where the main transverse commissure can be seen. Anterior is to the left. **D**. Serial transverse sections (from anterior to posterior), reconstructed from the confocal sections of **C**. Abbreviations: *alnc*, accessory lateral nerve cord; *arc*, anterior ring commissure; *esr*, external sucker ring; *isr*, internal sucker ring; *lg*, lateral ganglia; *lrc*, lateral rostellar connectives; *mlnc*, main lateral nerve cords; *mnc*, medial nerve cords; *mrc*, medial rostellar connectives; *mtc*, main transverse commissure; *plg*, postero-lateral ganglia; *prc*, posterior ring commissure; *rr*, rostellar ring; *rc*, coronae of rostellar ring projections; *x*, x-commissure. Bars represent 50 μm.

**Figure 7:**
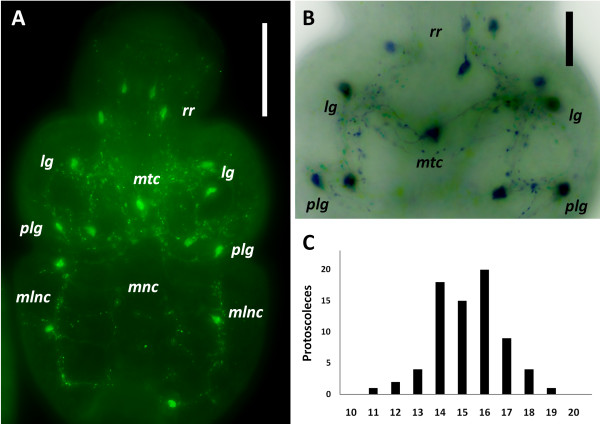
**5-HT-IR in the protoscolex. A**. General view of the protoscolex. **B**. Detail of the scolex (different specimen). **A** and **B** are merges of several focal planes of epifluorescence microscopy, anterior is to the top. **C**. Histogram showing the frequency of the abundance of 5-HT-IR nerve cells per protoscolex. Abbreviations are as in Figure 6. Bars represent 50 μm in **A**, 20 μm in **B**.

The nervous system of the protoscolex was reconstructed from confocal sections of whole mount material and minor details were obtained from cryosections. AcTub-IR revealed the perinuclear cytoplasm and the neurites of nerve cells, as well as the body and cilia of the flame cells (Figure 5), [Additional file 7]. Because of their morphology and distribution in the nervous system, we conclude that most of the cells labeled by AcTub-IR in the protoscolex, excluding the flame cells, are nerve cells. The flame cells possessed thin AcTub-IR protrusions projecting from the cell bodies, that sometimes appeared to contact the nerve cords. They were nonetheless easily distinguished from nerve cells thanks to their characteristic morphology and by the co-labeling with phalloidin at the base of the flame. A large number of nerve cells were detected by this method, resulting in exquisite detail, but at the same time obscuring in some instances the general layout of the nervous system. The FMRFa-IR, on the other hand, strongly stained all the main and minor nerve tracts in the scolex, the nerve cords and connecting commisures, as well as several minor projections from the nerve cords, giving the best results for the reconstruction of the general layout of the nervous system, although it was very difficult to recognize the perikarya of individual nerve cells (Figure 6), [Additional file 8]. The pattern of RYa-IR was essentially indistinguishable to that obtained by FMRFa-IR, and the pattern or FVa-IR was similar, except that IR was typically low in the nerve cords as compared to the scolex [Additional file 9]. Finally, 5-HT-IR identified a small subset of nerve cell bodies and their neurites, that follow the main nerve tracts, the nerve cords, and some minor tracts and projections (Figure 7). In combination, these markers allowed for a detailed description of the nervous system of the protoscolex.

### Neuroanatomy

The nervous system in the scolex of the protoscolex of *E. multilocularis* is very similar to that of the adult stage of *E. granulosus*. All the main elements observed by 5-HT IHF and/or acetylcholinesterase HC [16,17] were revealed by AcTub and FMRFa IHF, as well as some minor elements and details not previously described.

Two lateral ganglia are situated in the scolex at the level of the anterior border of the suckers. These contain numerous AcTub-IR nerve cell bodies, but it was not possible to distinguish a distinct neuropile within the ganglia, probably due to their small size. These ganglia are connected by a main transverse commissure and by an anterior ring commissure; the main transverse commissure displayed relatively low AcTub-IR, and in some FMRFa and AcTub IHF preparations it appeared to be formed by two individual commissures. From each lateral ganglion, two connectives are emitted towards a rostellar nerve ring at the base of the rostellar pad. These connectives are very closely juxtaposed, and only distinguishable by reconstruction from confocal sections. One pair of dorsal and one pair of ventral medial connectives also connect the anterior ring commissure to the rostellar ring and are the continuations of the minor medial nerve cords (see below). Each of these dorsal and ventral nerve pairs are further connected midway to the rostellum by a short commissure. Finally, a pair of dorso-ventral connectives, termed the ‘X-commissure’ by Shield [21], directly connect the dorsal and ventral medial nerve cords to the main transverse commissure, resulting in a characteristic cross as seen in cross-sections (Figures 6B, 6D).

The rostellar nerve ring itself appears as a smooth FMRFa-IR ring and has numerous AcTub-IR bipolar nerve cell bodies, that give rise to two coronae of long neurites innervating the rostellum. The neurites of the external corona surround the rostellar pad and reach the apex of the rostellum where the hooks are inserted (this region was denominated the ´rostellar cone´ by Galindo et al. [24] , and is probably the precursor of the so-called “rostellar gland” of the adult, composed by modified tegumental cells with secretory properties [25]). The internal corona is composed of neurites that reach and presumably traverse into the rostellar pad. Within the rostellar pad itself few AcTub+ neurites or cell bodies are visible. There seemed to be no indication of distinct lateral rostellar ganglia *sensu* Fairweather et al. [17], but rather the whole rostellar nerve ring seemed to be ganglionic in nature.

To the posterior, the lateral ganglia emit two thick connectives towards the postero-lateral ganglia, located at the same level as the posterior border of the suckers. By FMRFa-IR, the lateral ganglia, the thick connectives and the postero-lateral ganglia appear as a continuous mass of nervous tissue. The postero-lateral ganglia are connected to each other by a posterior ring commissure. The two main lateral nerve cords are born from the postero-lateral ganglia, and each of them is accompanied by a dorso-lateral and a ventro-lateral minor nerve cord, that are born at the point where the posterior ring commissure fuses with the sucker nerve ring (see below; Figure 6A, 6B; Additional file 8).

Each sucker is surrounded at its base by an external nerve ring, that fuses at its medial margin with the medial nerve cords and at its anterior and posterior margins with the anterior and posterior ring commisures. The sucker nerve ring has numerous AcTub+ IR cells projecting neurites that either surround the body of the sucker and result in nerve terminals at its opening, or go deep into the body of the sucker and connect to an internal sucker nerve ring. AcTub-IR nerve cell bodies are also present in this ring (Figure 5C), but only neurites are labeled by 5-HT-IR and FMRFa-IR. They appear to be bipolar, and extend long neurites towards the surface of the sucker.

In the posterior body, the five pairs of longitudinal nerve cords are connected by transverse ring commissures, forming a typical orthogonal plan [26,27], although the neurites connecting each pair of adjacent nerves are not completely aligned to each other. Along the main nerve cords, the bodies of nerve cells can be distinguished by AcTub-IR, FMRFa-IR, RYa-IR and 5-HT-IR (Figures 6A, 7A, Additional file 9C and data not shown). From these cell bodies neurites extend towards the subtegumental region, where they form a peripheral plexus. Among the AcTub-IR neurites, some of their terminals are located in the subtegumental region, presumably innervating the subtegumental muscles, while others traverse the tegument and are therefore probably sensory terminals (such as those previously described by TEM in *E. granulosus* adults by Morseth [28]; Figure 5E). There are many medial AcTub-IR neurites, that make the medial nerve cords untraceable with AcTub-IR (but they are readily distinguished by IR to the other antibodies). In the scolex, the nerve terminals of the peripheral plexus are very densely distributed (Figure 5A).

Throughout this description, 5-HT-IR was scarcely mentioned because of the similarity to previous results in *E. granulosus* adults [17]. An important distinction, however, lies in the number of 5-HT-IR nerve cells observed. We observed that although the positions of the 5-HT-IR cells were relatively fixed, the number of cells in each position and the total number in each protoscolex showed some variation. This variation occurred in completely mature protoscoleces, as judged by the morphology of the suckers and the rostellum, and also by the low levels of cell proliferation as determined by 5-ethynyl-2′-deoxyuridine (EdU) incorporation assays (U. Koziol and K. Brehm, unpublished results; see [29]). 5-HT-IR nerve cell bodies were located in the rostellar nerve ring (mode = 3, range = 2–4), in each lateral ganglion (mode = 1, range = 1–2), on the region of the anterior commissure (mode = 1, range = 0–3), in each postero-lateral ganglion (mode = 2, range = 1–3), on the main lateral nerve cords (mode = 2, range = 1–3) and rarely on the medial nerve cords (mode = 0, range = 0–2) (Figure 7). More rarely, we observed ectopically positioned 5-HT-IR nerve cell bodies (e.g. between nerve cords). The total number of 5-HT-IR cells was therefore variable, with an average of 15.2 cells per protoscolex (n = 74), and the majority had between 14 and 17 5-HT-IR cells (Figure 7C). This was the case for both GH09, an isolate kept by intraperitoneal serial passage in *M. unguiculatus* for 3 years (average = 15.3 5-HT-IR cells / protoscolex), as for J2012, that had only been passaged for 6 months (average = 15.2 5-HT-IR cells / protoscolex; n = 37 for each isolate). This indicates that this variation is a typical feature of the *E. multilocularis* protoscolex and not an abnormal characteristic of a specific laboratory isolate.

### The development of muscle fibers and the nervous system in the protoscolex

#### IR patterns obtained with each antibody during development

Phalloidin staining revealed the development of the muscle fibers throughout protoscolex development. In the case of the nervous system, 5-HT-IR was detectable from early stages of development, clearly labeling the cell bodies of nerve cells, and later the projections from these cells. FMRFa-IR was detectable either at the same stages of development as 5-HT, or usually slightly later, showing similar patterns. Finally, AcTub-IR appeared relatively late during development, only in those regions of the nervous system that could be previously detected by the other antibodies. However, a greater number of nerve cells could be detected at this point with this antibody.

### Developmental stages

**Figure 8:**
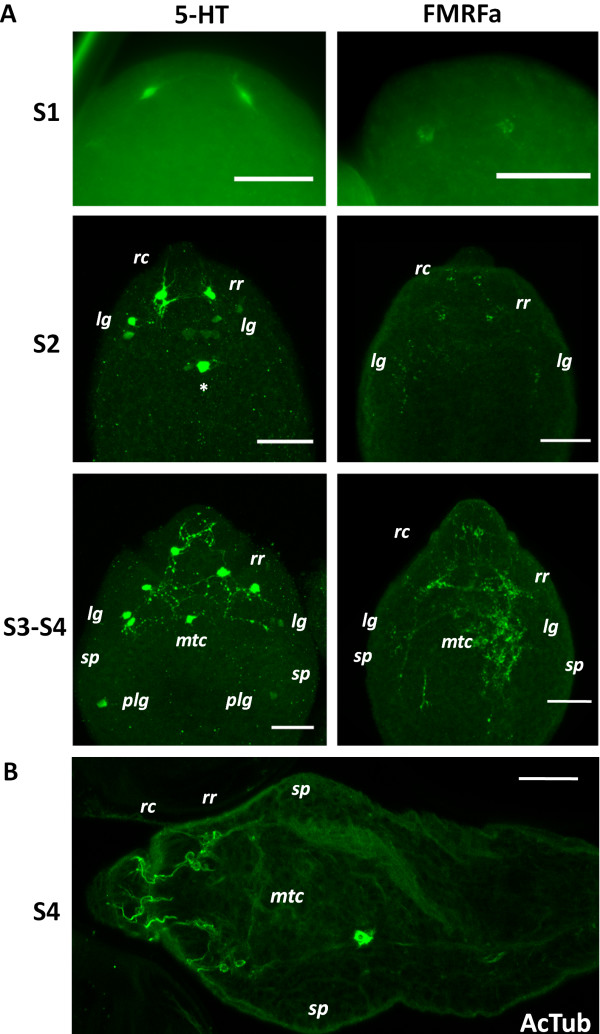
**Development of the protoscolex nervous system. A**. 5-HT-IR and FMRFa-IR in the early stages of protoscolex development. Abbreviations are as in Figure 6 and *sp*, sucker primordium. The asterisk marks a 5-HT-IR cell in an unusual position, perhaps in the developing *mtc*. Note also some supernumerary 5-HT-IR cells with low signal levels next to the lateral ganglia. **B**. AcTub-IR in late protoscolex development. S1, S2, S3, S4 are stages 1 to 4, respectively. Bars represent 20 μm.

We have adopted the staging system of Leducq and Gabrion [7] for describing the development of the protoscolex. In stage 1, the initial protoscolex bud appears in the wall of the brood capsule, without any external distinctive features. Already at this stage, there is a well defined grid of longitudinal and circular subtegumental muscles [Additional file 10], that continue into the wall of the brood capsule, and several transverse muscle fibers. It is at this stage that 5-HT-IR appears, as a pair of 5-HT positive cells close to the tip of the bud, without any projections except that they are connected to each other by a short commissure (Figure 8A). Rarely, faint FMRFa-IR also appears in a pair of cells in a similar position (Figure 8A). These cells mark the beginning of the development of the rostellar nerve ring. The development of the nervous system then proceeds towards the posterior, resulting in a developmental gradient along the antero-posterior axis.

In stage 2, the rostellar primordium becomes apparent at the tip, composed of an apical rostellar bulb, that will differentiate into the muscular rostellar pad, and a fold (the prebulbar region) at its base. Some of the deep longitudinal muscle fibers can already be seen at this stage. [Additional file 11]. The nerve cells in the developing rostellar ring form anterior projections towards and into the rostellar bulb, and in some cases 5-HT-IR and FMRFa-IR cells can be seen at this and later stages within the rostellar bulb (however, these probably become relocated later during development since no such cells were observed in the mature rostellar pad). At the same time posterior projections are formed towards the developing lateral ganglia, in which some 5-HT-IR cells with varying labeling intensity can already be seen, already connected by the main nerve commissure (Figure 8A), [Additional file 11]. The longitudinal nerve cords cover part of the antero-posterior axis. Sometimes supernumerary 5-HT-IR cells or positive cells with atypical positions can be seen (Figure 8A). The fate of these cells is not clear; presumably these are eliminated or relocated later in development. The FMRFa-IR pattern closely matches the 5-HT-IR, except that it extends more posteriorly in the developing longitudinal nerve cords (Figure 8A). No AcTub-IR is usually seen at this stage.

In stages 3 to 4, the region that will become the scolex becomes distinct from the posterior body, and cells begin to accumulate at the site where the suckers will be developed. At the same time, the histogenesis of the hook begins at the side of the prebulbar fold. At these stages, the rostellar ring and its coronae, the lateral ganglia, the main transverse commissure and the anterior ring commissure are clearly evident by 5-HT and FMRFa IHF, and weakly 5-HT-IR cells can be seen at the position of the postero-lateral ganglia (Figure 8A). The main lateral nerve cords are seen extending posteriorly and the anterior portion of the medial nerve cords is also distinct. At this point, AcTub-IR brightly labels the cells of the rostellar nerve ring and the coronae of rostellar projections, directed towards the bulb (internal corona) and the prebulb (external corona) (Figure 8B).

In stages 5 to 6, the development of the suckers and their associated muscles and nerve rings proceeds. At the same time, the folds of the rostellar prebulb, carrying the developing hooks, grow and engulf the rostellar bulb, coalescing over it. Therefore, the rostellar bulb becomes sub-apical, with the hooks and the prebulb covering it as is found in the mature rostellum. At this point minor connectives and commissures in the scolex are developing, and the postero-lateral ganglia and the posterior ring commissure are well formed. FMRFa-IR labels the main nerve cords all the way towards the posterior of the protoscolex soma; 5-HT-IR usually labels the cords only to a more anterior position, but positive cells can already be found on the main nerve cords. Finally, at stage 7 the development is complete and the scolex lies invaginated within the posterior body.

## Discussion

This work represents the first detailed description of the nervous and muscle systems in the development of the post-onchosperal larvae of *Echinococcus*, including the first description, to the best of our knowledge, of nervous elements in the germinal layer of the metacestode. The presence of muscle fibers and nerve cells in the germinal layer of the metacestode is intriguing, since this larval stage is a non-motile cyst. However, possible functions for these systems can be hypothesized. In the case of the muscle system, the accumulation of circular fibers around the invaginating brood capsule suggests that they could be involved in the mechanism of invagination and in the constriction of the stalk. Furthermore, the muscle layer in the cyst wall is continuous to that of the brood capsule and the protoscolex. In the case of the nervous system, although the projecting neurites of the nerve cells are in close apposition to the muscle fibers, a myoregulatory role seems improbable, given the lack of motility and the absence of serotoninergic and cholinergic elements (which are the best characterized myomodulatory neurotransmitters in flatworms [22,30]). Furthermore, this nervous system is independent from that of the developing protoscolex, suggesting that it would not serve as a scaffold for the development of the protoscolex nervous system. An attractive hypothesis is that it could represent a neuroendocrine system, secreting factors that regulate the development of the metacestode. Relatedly, possible neuroendocrine secretory terminals have been described at the ultrastructural level in the nervous system of cestodes [31], and in free living flatworms there are several lines of evidence suggesting a role of the nervous system in regulating cell proliferation and pattern formation during asexual reproduction and regeneration [32,33]. In other systems, muscle fibers or myofibroblasts can also be a source of growth factors stimulating stem cell proliferation and maintenance in superjacent epithelia [34,35]. If such a role was demonstrated for the neuromuscular system in *Echinococcus* cysts, it would open new areas for drug research targeting these signaling systems, offering a potential link to many present efforts targeting the neuromuscular system in trematodes [36].

The nervous and muscle systems in *E. multilocularis* are probably homologous to those present in the motile bladder tissue from *Taenia* spp., although modified because of the lack of motility in *Echinococcus* cysts. Indeed, the limited reports regarding the nervous system in the bladder of *Taenia* spp. indicate that RFamide-IR and acetylcholinesterase positive (AChE+) fibers are present [37,38]. Furthermore, we have confirmed the presence of FMRFa-IR, AcTub-IR and AChE+ elements in the bladder tissue of *in vitro* cultured *Taenia crassiceps*, together with a well organized grid of subtegumental longitudinal and circular muscles [Additional file 12].

In another publication [39] we performed a bioinformatic search for neuropeptide-encoding genes in the genomes of *E. multilocularis* and other cestodes, finding several genes that showed significant expression in the germinal layer (as determined by high throughput RNA sequencing and RT-PCR). It is thus possible that some of these neuropeptides are expressed in the nerve net of the germinal layer.

Surprisingly, there is no AcTub-IR during the early development of the protoscolex nervous system, which is at odds with the occurrence of AcTub-IR in pioneer neurons during the development of many metazoans [40-43]. It seems that in the case of *E. multilocularis*, tubulin-α acetylation occurs only at late stages of neurogenesis. Interestingly, 5-HT and FMRFa-IR were detected from very early time points, even in young nerve cells with few short projections. Similar results have been described in the development of other invertebrates [44-46], and the early expression of neurotransmitter molecules could have regulatory roles during the development of the nervous system [47-49].

This description of the nervous system in the scolex is the most detailed to date, benefiting from the use of several different markers in combination with confocal microscopy. In particular, the use of AcTub-IR resulted in great detail, and allowed us to identify large numbers of nerve cell bodies not previously detectable with 5-HT-IR, AChE HC or IR against mammalian neuropeptides [16-18]. Prominent examples of this include the rostellar ring, which appears as fully ganglionic by AcTub-IR (whereas it appeared to be composed of two individual ganglia by 5-HT-IR) and the inner sucker nerve ring, that has numerous AcTub-IR cell bodies but that was only described as a network of nerve processes in previous studies [17,18]. On the other hand, FMRFa-IR gave the best results for the reconstruction of the general layout of the nervous system, and for tracing the main commisures and connectives. Indeed, it allowed the detection of all nervous elements previously described from 5-HT-IR and/or AChE HC, together with fine details not previously described [Additional file 13].

Once the development of the nervous system is complete in the protoscolex, its morphology is already similar to that of the scolex of the developed adult, with no major rearrangements produced during adult development. This is probably related to the fact that the scolex, once evaginated, must be fully functional to avoid being dispelled from the host. However, the number of 5-HT-IR cells in the scolex of adult *E. granulosus* is higher, and has been described as fixed for each region (e.g., exactly five 5-HT-IR cells have been described in the postero-lateral ganglia, 3 in the rostellar ring, etc. [17]). Therefore, assuming that the nervous system of the adult of *E. multilocularis* is identical to that of *E. granulosus*, new nerve cells must be incorporated into the nervous system of the scolex during adult development (as has been described for example for peptidergic neurons of *Hymenolepis diminuta*[50]). The final numbers of 5-HT-IR cells might become fixed in adults as compared to the protoscolex, but there is little information about the constancy in the number and position of individually identifiable neurons in flatworms in general [26,40]. The general structure of the nervous system in the scolex of *Echinococcus* is also remarkably similar to classical descriptions of the related *Taenia* spp. [38,51], although the scolex in these species is at least one order of magnitude larger. It is also worth mentioning the similarities observed in the development of the scolex of *Echinococcus* spp. ([8]; this work) and that of *Taenia* spp. [52-54]. In both cases the rostellar bulb and prebulb are the first signs of differentiation appearing on a thickened scolex primordium in the brood capsule (*Echinococcus*) or bladder wall (*Taenia*). Later, as the prebulb carrying the hooks grows and engulfs the bulb, the primordia of the suckers are formed.

The highly detailed description of the nervous system in these larval stages is a necessary first step towards future studies regarding the function of the nervous system, and of particular neurotransmitters, neuropeptides and their signal transduction systems in the biology and development of *E. multilocularis*. Furthermore, the use of axenic *in vitro* cultivation systems for *E. multilocularis* metacestodes [55] provides the opportunity, for the first time, to study the mechanisms behind the peculiar developmental processes of these organisms, including their developmental plasticity, unusual asexual multiplication by protoscolex formation, and the continuous growth and exogenous budding of metacestodes, all of which are based on the presence of a totipotent population of stem cells (the so-called germinative cells or neoblasts) [2,56-58]. Also in this case, a careful description of developmental sequences such as the development of the neuromuscular system described herein is an essential first step.

## Conclusions

*E. multilocularis* is an important human parasite and a recently emerging model system to study host-dependent parasite development *in vitro*[59]. We herein provide the most detailed description to date of the morphology of the nervous system of protoscoleces throughout development. We found a remarkable complexity of the protoscolex nervous system, comparable to that observed in many adult cestodes. The data we provide will be important for future studies on parasite developmental plasticity and functional and evolutionary analyses on cestodes in general, and taeniids in particular. Furthermore, we demonstrate for the first time that the cystic metacestode stage, which grows tumor-like within the intermediate host, contains a nerve net with peptidergic elements. Although the function of this nerve net cannot yet be clearly determined, we suggest that it could act as a neurendocrine system that regulates parasite development within the host. As such, it could be a potential target for the development of novel anti-parasitics. Using the information provided by our study, and the recently determined *E. multilocularis* genome sequence [42], we are currently characterizing parasite neuropeptides that are expressed in the metacestode in order to determine their function in parasite physiology.

## Materials and Methods

### Parasite culture and fixation

Parasite isolates were maintained by serial intraperitoneal passage in *Meriones unguiculatus* as previously described [55]. Isolates were originally obtained from accidental infections of Old World Monkeys in a breeding exclosure [60], and had been passaged at the time of this study for between six months and four years. All experiments were carried out in accordance with European and German regulations on the protection of animals (Tierschutzgesetz). *In vitro* co-culture of parasite vesicles with rat Reuber hepatoma cells was performed as previously described [55]. Protoscoleces were isolated from *in vivo* parasite material and either activated by successive treatments with pepsin at low pH and taurocholate [61] , or alternatively set into culture in DMEM (Gibco) plus 10% fetal calf serum (Biochrom) for 12 hours, that also resulted in significant scolex evagination. Parasite vesicles obtained *in vitro*[55] were gently opened using a syringe tip in order to allow the entry of the fixative and other reagents during immunohistofluorescence. The material analyzed from *in vivo* cultures consisted of small pieces of metacetode tissue surrounded by host liver tissue. All samples for IHF were fixed in 4% paraformaldehyde (PFA) buffered in phosphate buffer saline (PBS), for 1 to 4 hours at room temperature.

### Antibodies

We used a suite of antibodies typically employed for the description of the nervous system in diverse invertebrates:

1) Anti-acetylated tubulin-α (AcTub), mouse monoclonal antibody (Santa Cruz, clone 6-11B-1; diluted 1:100; [62]). Tubulin-α acetylation is a post-translational modification that occurs in highly stable microtubuli, mainly occurring in the neuronal cytoskeleton (neurotubuli) and in cilia and flagella. Because it is able in principle to label all nerve cells independently of their neurotransmitter, AcTub immunoreactivity (IR) has been used to describe in detail the complete nervous system of numerous invertebrates, including free living flatworms and onchospheres [40-42,59,63-65]. Furthermore, it has been used to study the development of the nervous system in both vertebrates and invertebrates, since many pioneer axons show AcTub-IR [40-43,66].

2) Anti-FMRFamide (FMRFa), rabbit polyclonal antibody (Immunostar, diluted 1:300). Antibodies against the neuropeptide FMRFa have also been used extensively for the characterization of invertebrate nervous systems. It is acknowledged that anti-FMRFa antibodies, and in particular the one used in this and other studies, can recognize most neuropeptides containing a C-terminal RFamide motif [21,64,67,68].

3) Anti-RYamide (RYa; 1:250 dilution) and Anti-FVamide (FVa; 1:125 dilution), rabbit polyclonal antibodies, kindly provided by Markus Conzelmann (Max Planck Institute for Developmental Biology, Tübingen, Germany). These antibodies recognize small amidated dipeptide motifs frequently found in neuropeptides of invertebrates, and are highly specific [69]. For instance, the anti-RYa antibody does not cross- react with neuropeptides with an RFamide motif. Anti-FLamide was also used but no IR was found in *E. multilocularis* (data not shown).

4) Anti-5-HT, rabbit polyclonal antibody (Immunostar, diluted 1:300). Antibodies against 5-HT have been extensively used to characterize the serotoninergic components of the nervous system in invertebrates in general and flatworms in particular (see for example [17,18,22,64]).

### Immunohistofluorescence in whole-mounts (WMIHF) and in cryosections

We followed the WMIHF protocols described by Collins et al. [70] for *Schistosoma mansoni*, with some modifications. After fixation, samples were washed three times for 10 min with PBS with 0.3% Triton X-100 (PBS-T). After this, permeabilization was achieved by either A) 20 min incubation in PBS with 1% sodium dodecyl sulfate (SDS) for metacestode vesicles or B) a 5 min treatment with proteinase K (Fermentas, 2 μg/ml in PBS-T plus 0.5% SDS), followed by re-fixation with 4% PFA buffered in PBS for 10 min at room temperature (proteinase K treatment was essential for protoscolex WMIHF). After permeabilization, samples were washed three more times with PBS-T and blocked for 2 h in PBS-T with 3% bovine seralbumin (BSA, Sigma-Aldrich) and 5% normal sheep serum (Sigma-Aldrich). Incubation with the primary antibody, diluted in PBS-T with 3% BSA, was carried overnight at 4°C. Then, samples were washed five times for 30 min with PBS-T, and incubated overnight with the FITC conjugated secondary antibody (donkey anti-mouse or donkey anti-rabbit as required, Jackson Immunoresearch) at 4°C. Finally the samples were washed five times for 30 min with PBS-T, and co-stained with 4',6-diamidino-2-phenylindole (DAPI) and TRITC-conjugated Phalloidin (Sigma-Aldrich) as previously described [71]. Negative controls lacking the primary antibody were performed, which showed no signal. Cryosections were prepared as previously described [57], and processed for IHF with a similar protocol, except that no detergent was added to PBS for the washes, permeabilization was achieved using 0.1% Triton X-100 in PBS, and all incubation times were reduced. Samples were analyzed by confocal microscopy (Leica TCS SP5) and by epifluorescence microscopy (Zeiss Axio Imager.Z1).

### Acetylcholinesterase Histochemistry (AChE HC)

AChE HC was carried out as described by the direct method of Karnovsky and Roots [72].

### Transmission electron microscopy

Metacestode vesicles obtained after 3 months of *in vitro* culture of the GH09 isolate (containing brood capsules) were fixed, processed and analyzed by TEM, essentially as previously described [73].

## Competing interests

The authors declare that they have no competing interests.

## Authors’ contributions

UK carried out or participated in all experiments. UK and KB designed the study and drafted the manuscript. GK designed and interpreted the results of TEM studies. All authors read and approved the final manuscript.

## Supplementary Material

Additional file 1 **Unusual flame cell in the germinal layer of *Echinococcus multilocularis.*** Confocal microscopy, section of *in vivo* cultured material. The bar represents 10 μm. Click here for file

Additional file 2 **AChE histochemistry in *Echinococcus multilocularis. *A**. Double labeling of AChE HC and AcTub-IR; note the single AChE HC positive cell without any projections (asterisk), and negative to AcTub-IR. AcTub-IR cell bodies are indicated by arrows. The bar represents 50 μm. **B**. AChE HC reaction in the nervous system of the protoscolex. Click here for file

Additional file 3 **Transmission electron microscopy of the germinal layer of *Echinococcus multilocularis.*** Nerve projections (**A**, **B**, **C**, arrows) and a putative nerve cell body (**C**, asterisk) are highlighted in red. Abbreviations: *mc*, mitochondria; *nu*, nucleus; *tu*, microtubules; *ve*, vesicles. Bars represent 1 μm. Click here for file

Additional file 4 **Early brood capsule.** Serial confocal sections of an early developing brood capsule from *in vivo* cultured material of *Echinococcus multilocularis* stained for AcTub-IR (green), Phalloidin (red) and DAPI (blue). Click here for file

Additional file 5 **Stalked brood capsule.** Serial confocal sections of a brood capsule with a stalk from *in vivo* cultured material of *Echinococcus multilocularis* stained for Phalloidin (red) and DAPI (blue). Click here for file

Additional file 6 **Brood capsule with developing protoscolex.** Serial confocal sections of a brood capsule with a protoscolex bud in a whole-mount *in vitro* cultured cyst of *Echinococcus multilocularis*, stained for AcTub-IR (green), Phalloidin (red) and DAPI (blue). Click here for file

Additional file 7 **AcTub-IR in the protoscolex of *Echinococcus multilocularis.*** Serial confocal sections of a whole-mount specimen. Click here for file

Additional file 8 **FMRFa-IR in the protoscolex of *Echinococcus multilocularis.*** Serial confocal sections of a whole-mount specimen stained for FMRFa-IR (green), Phalloidin (red) and DAPI (blue). Click here for file

Additional file 9 **FVa-IR and RYa-IR in the protoscolex of *Echinococcus multilocularis. *A**. Developing protoscolex, FVa-IR (whole-mount). **B**. Transverse section of the scolex, FVa-IR. **C**. Mature protoscolex, RYa-IR (whole-mount). **D**. Section of developing protoscolex, RYa-IR. Abbreviations are as in Figures 4 and 6, and *cns*, central nervous system; *px*, subtegumental plexus. Bars represent 50 μm in A, C and D, and 25 μm in B. Click here for file

Additional file 10 **Subtegumental muscle fibers in a stage 1 protoscolex bud of *Echinococcus multilocularis.*** Serial confocal sections of a whole-mount protoscolex bud partially surrounded by the remains of the brood capsule, stained with Phalloidin. Click here for file

Additional file 11 **5-HT-IR in a stage 2 developing protoscolex of *Echinococcus multilocularis.*** Serial confocal sections of a whole-mount specimen stained for 5-HT-IR (green) and Phalloidin (red). Click here for file

Additional file 12 **Muscle and nervous systems in *Taenia crassiceps *bladder tissue cultured *in vitro.*** This is a laboratory strain passaged in *M. unguiculatus*. It multiplies by budding but is unable to produce scoleces, generating only bladder tissue. Samples were processed identically to the *E. multilocularis* cysts. **A**. Phalloidin staining; note the longitudinal and circular muscle fibers (whole-mount). **B**. AcTub-IR in the subtegumental layer; inset shows the flame cells in detail (whole-mount). **C**. FMRFa-IR in the subtegumental layer (whole-mount). **D**. AChE HC (section). Bars represent 200 μm in A, 50 μm in B and C, 20 μm in the inset in B. Click here for file

Additional file 13 Comparison of the description of the protoscolex nervous system in this and in previous investigations. Click here for file
