# Supplementary figures and images for: Anatomy and development of the larval nervous system in Echinococcus multilocularis
Source: Front Zool. 2013 May 4;10:24. doi: 10.1186/1742-9994-10-24 (PMC3658878; doi:10.1186/1742-9994-10-24)

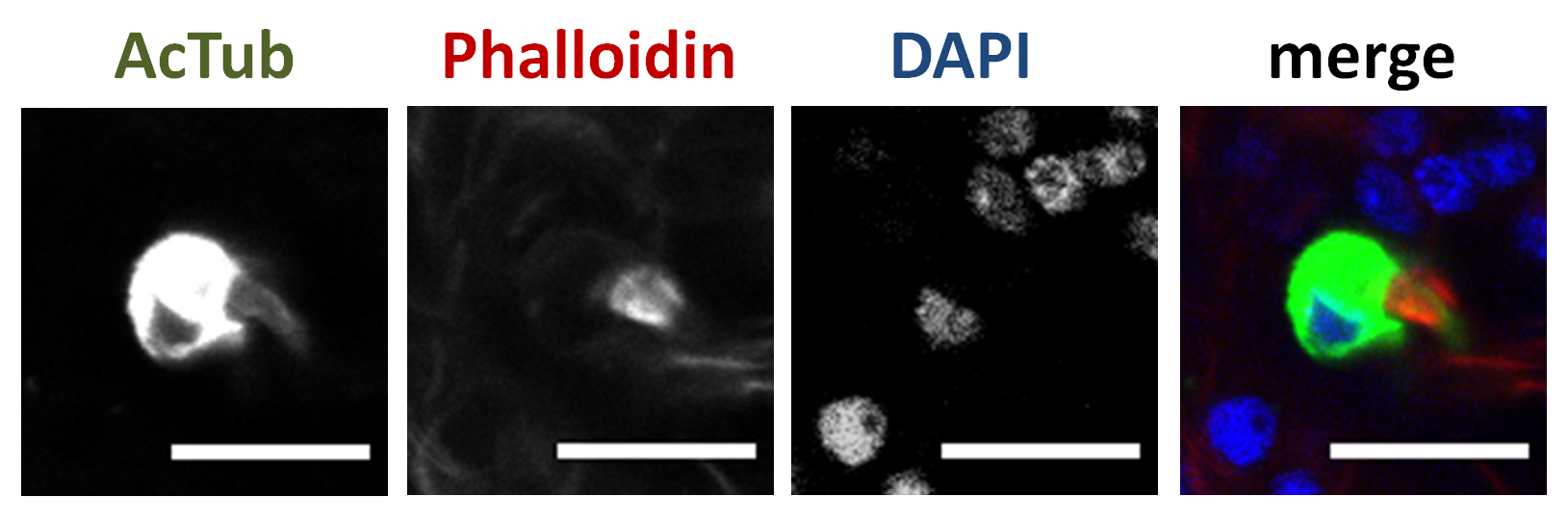

Supplement: Additional file 1 — Unusual flame cell in the germinal layer of Echinococcus multilocularis. Confocal microscopy, section of in vivo cultured material. The bar represents 10 μm. [file 1742-9994-10-24-S1.tiff]

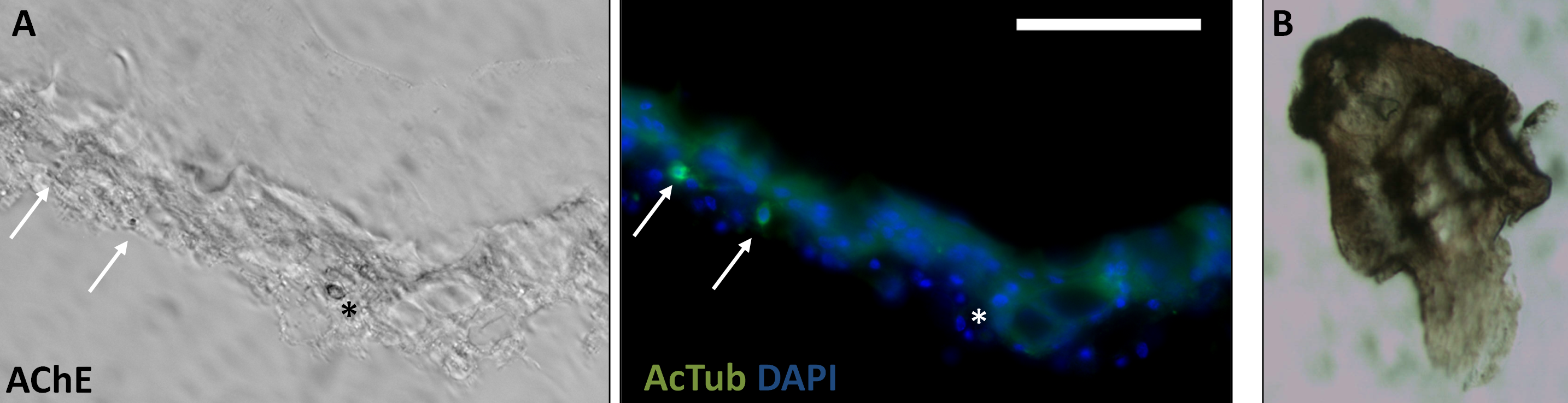

Supplement: Additional file 2 — AChE histochemistry in Echinococcus multilocularis. A. Double labeling of AChE HC and AcTub-IR; note the single AChE HC positive cell without any projections (asterisk), and negative to AcTub-IR. AcTub-IR cell bodies are indicated by arrows. The bar represents 50 μm. B. AChE HC reaction in the nervous system of the protoscolex. [file 1742-9994-10-24-S2.tiff]

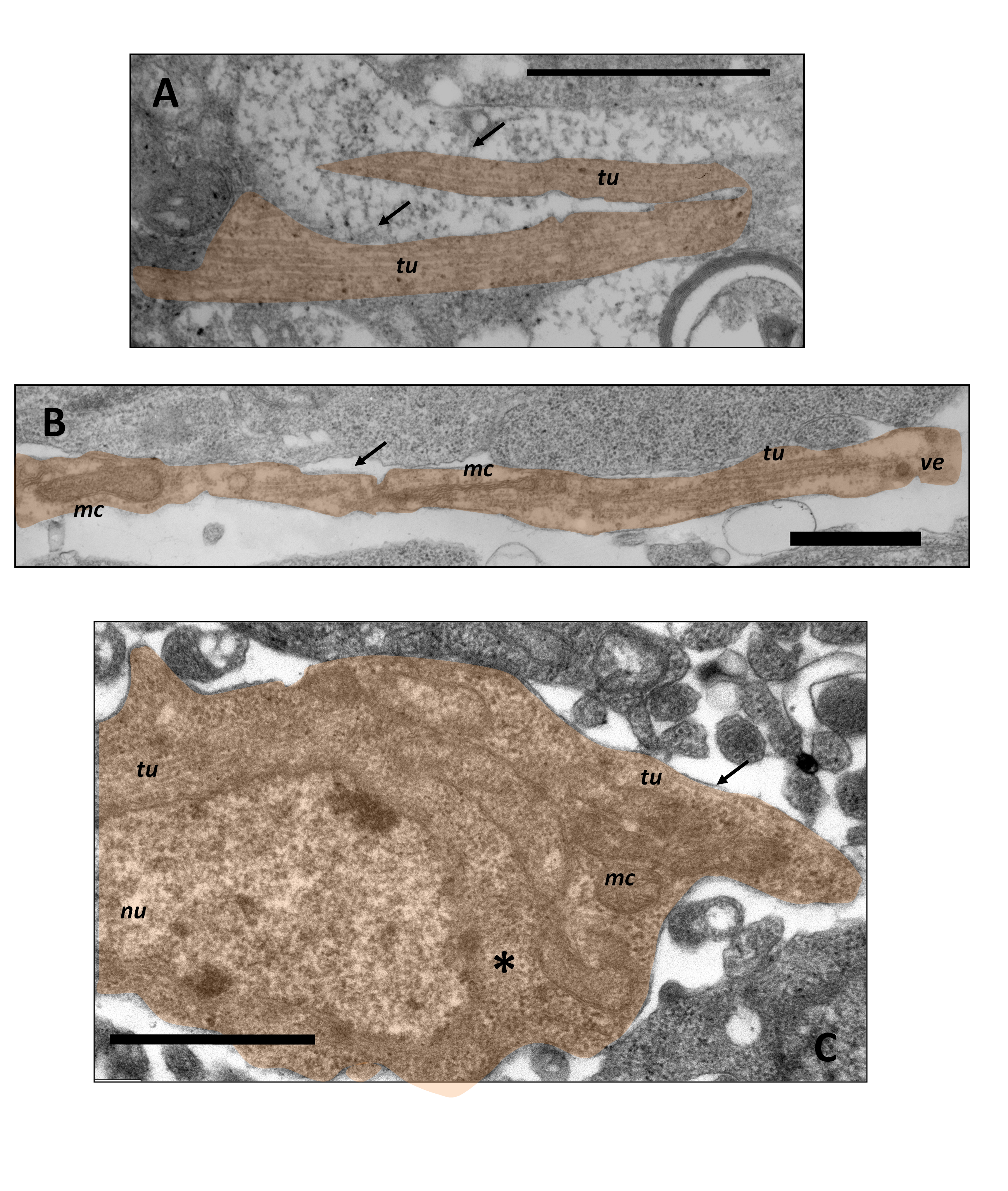

Supplement: Additional file 3 — Transmission electron microscopy of the germinal layer of Echinococcus multilocularis. Nerve projections (A, B, C, arrows) and a putative nerve cell body (C, asterisk) are highlighted in red. Abbreviations: mc, mitochondria; nu, nucleus; tu, microtubules; ve, vesicles. Bars represent 1 μm. [file 1742-9994-10-24-S3.tiff]

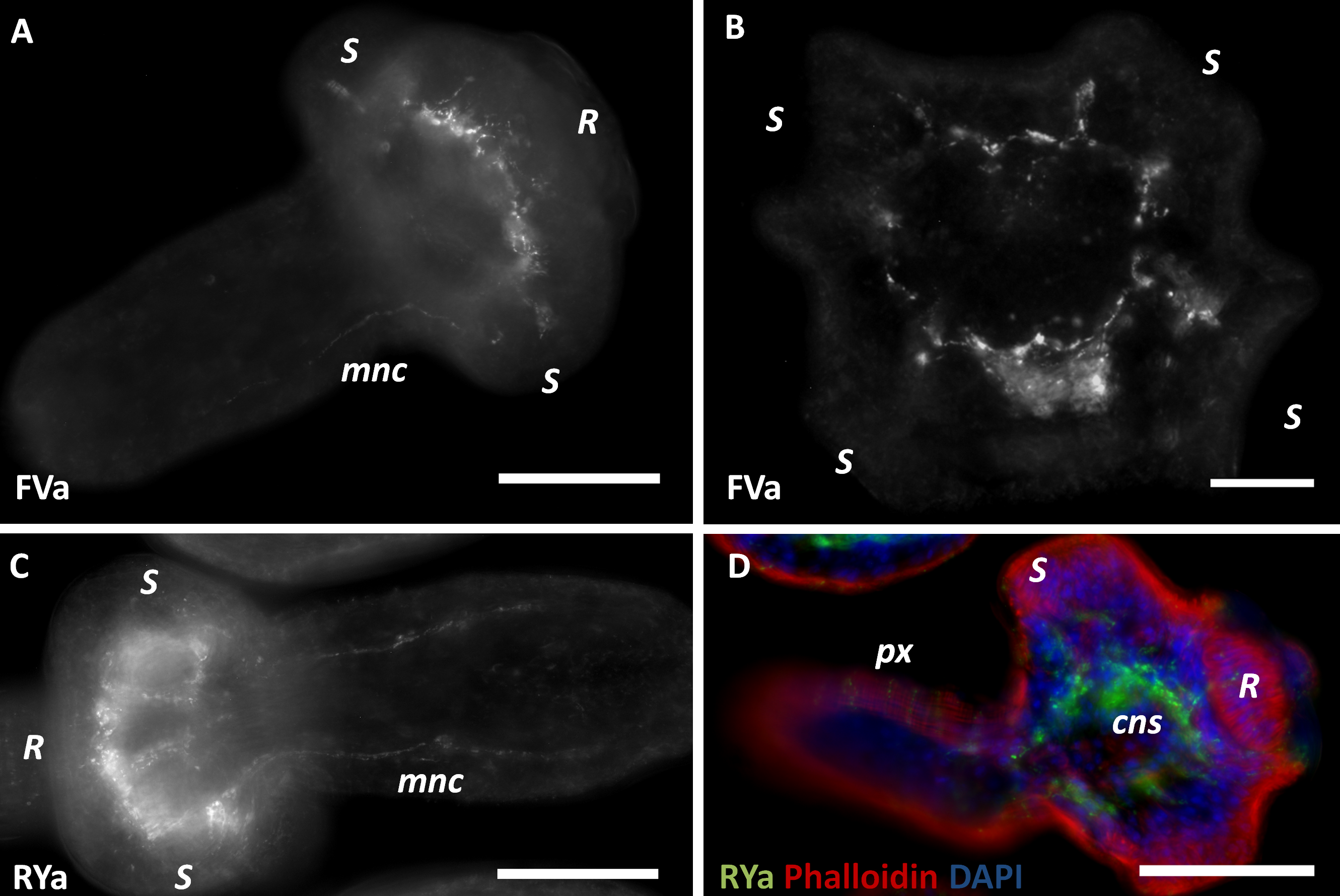

Supplement: Additional file 9 — FVa-IR and RYa-IR in the protoscolex of Echinococcus multilocularis. A. Developing protoscolex, FVa-IR (whole-mount). B. Transverse section of the scolex, FVa-IR. C. Mature protoscolex, RYa-IR (whole-mount). D. Section of developing protoscolex, RYa-IR. Abbreviations are as in Figures 4 and 6, and cns, central nervous system; px, subtegumental plexus. Bars represent 50 μm in A, C and D, and 25 μm in B. [file 1742-9994-10-24-S9.tiff]

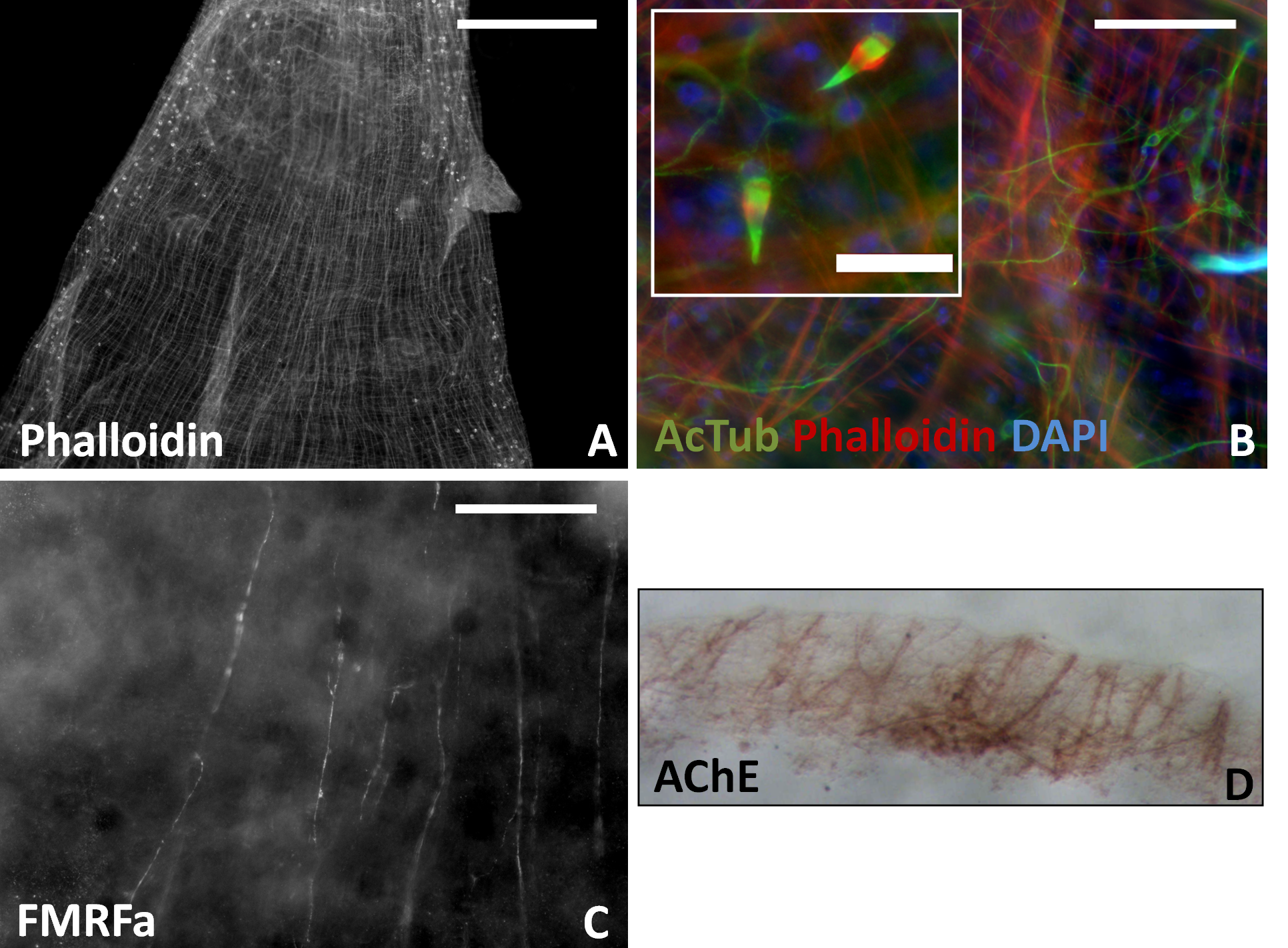

Supplement: Additional file 12 — Muscle and nervous systems in Taenia crassiceps bladder tissue cultured in vitro. This is a laboratory strain passaged in M. unguiculatus. It multiplies by budding but is unable to produce scoleces, generating only bladder tissue. Samples were processed identically to the E. multilocularis cysts. A. Phalloidin staining; note the longitudinal and circular muscle fibers (whole-mount). B. AcTub-IR in the subtegumental layer; inset shows the flame cells in detail (whole-mount). C. FMRFa-IR in the subtegumental layer (whole-mount). D. AChE HC (section). Bars represent 200 μm in A, 50 μm in B and C, 20 μm in the inset in B. [file 1742-9994-10-24-S12.tiff]
