# Supplementary material for: Anatomy and development of the larval nervous system in Echinococcus multilocularis
Source: Front Zool. 2013 May 4;10:24. doi: 10.1186/1742-9994-10-24 (PMC3658878; doi:10.1186/1742-9994-10-24)
Supplement: Additional file 13 — Comparison of the description of the protoscolex nervous system in this and in previous investigations. [file 1742-9994-10-24-S13.doc]

|  | ***Echinococcus granulosus* adult**  **(Shield, 1969)**  **(AChE HC)** | ***Echinococcus granulousus* adult**  **(Brownlee *et al*., 1994)**  **(5-HT-IR)** | ***Echinococcus granulosus* protoscolex**  **(Fairweather *et al*., 1994)**  **(5-HT-IR)** | ***Echinococcus multilocularis* protoscolex**  **(this work)** |
| --- | --- | --- | --- | --- |
| **Rostellar ring and ganglia** | Present | Present, with three 5-HT-IR cells, in paired rostellar ganglia | Absent | Present, as a ganglionic ring of AcTub-IR cells with two to four 5-HT-IR cells. Compact ring observed with FMRFa-IR |
| **Coronae of nerves projecting from the rostellum** | Present, with elements both penetrating and surrounding the rostellar pad | Present, but only elements penetrating the rostellar pad were described | Absent | Present with elements both penetrating and surrounding the rostellar pad (Observable with all antibodies) |
| **Lateral rostellar connectives** | Present, simple | Present, simple | Present, simple | Present, double (distinguished as such by FMRFa-IR) |
| **Medial rostellar connectives** | Present | Absent | Absent? | Present |
| **Minor commissure between medial rostellar nerve pairs** | Absent | Absent | Absent? | Present (detectable by AcTub-IR, FMRFa-IR, and sometimes by 5-HT-IR) |
| **X-commissure** | Present | Absent | Absent? | Present (detectable by FMRFa-IR) |
| **Lateral ganglia** | Present | Present, with two 5-HT-IR cells | Present, with two 5-HT-IR cells | Present, with one or two 5-HT-IR cells |
| **Main transverse commissure** | Present | Present, with two 5-HT-IR cells (described as belonging to the lateral ganglia) | Present | Present (usually detectable as a double commissure by AcTub-IR and FMRFa-IR),with zero to three 5-HT-IR cells |
| **Anterior ring commissure** | Present | Present | Present | Present |
| **Postero-lateral ganglia** | Absent | Present, with five 5-HT-IR cells | Absent | Present, with one to three 5-HT-IR cells |
| **Posterior ring commissure** | Absent | Absent but “fine nerve processes” connecting the postero-lateral ganglia | Absent | Present, observable with all the antibodies |
| **External sucker nerve ring** | Absent? (described as a series of nerves from the ganglia and nerve cords?) | Absent? (described as a series of nerves from the ganglia and nerve cords?) | Present (described as a thin ring of varicose fibres) | Present, strongly labeled with FMRFa-IR |
| **Internal sucker nerve ring** | Present | Present, but only as a ring of neurites | Present, but only as a ring of neurites | Present, with AcTub-IR nerve cell bodies |
